# A Stretchable Alternating Current Electroluminescent Fiber

**DOI:** 10.3390/ma11020184

**Published:** 2018-01-24

**Authors:** Dan Hu, Xiuru Xu, Jingsheng Miao, Ori Gidron, Hong Meng

**Affiliations:** 1 School of Advanced Materials, Peking University Shenzhen Graduate School, Shenzhen 518055, China; 1501213864@sz.pku.edu.cn (D.H.); miaojs@pkusz.edu.cn (J.M.); 2 Institute of Chemistry, The Hebrew University of Jerusalem, Edmond J. Safra Campus, Jerusalem 91904, Israel; ori.gidron@mail.huji.ac.il

**Keywords:** stretchable fibers, scalable, electroluminescence device

## Abstract

Flexible, stretchable electroluminescent fibers are of significance to meet the escalating requirements of increasing complexity and multifunctionality of smart electronics. We report a stretchable alternating current electroluminescent (ACEL) fiber by a low-cost and all solution-processed scalable process. The ACEL fiber provides high stretchability, decent light-emitting performance, with excellent stability and nearly zero hysteresis. It can be stretched up to 80% strain. Our ACEL fiber device maintained a stable luminance for over 6000 stretch-release cycles at 50% strain. The mechanical stretchability and optical stability of our ACEL fiber device provides new possibilities towards next-generation stretchable displays, electronic textiles, advanced biomedical imaging and lighting, conformable visual readouts in arbitrary shapes, and novel health-monitoring devices.

## 1. Introduction

Smart electronic devices have attracted much attention due to the expectation that they can be easily stretched, compressed, bended, twisted, folded, and knotted. As a type of electronic device, providing an essential functionality, stretchable electroluminescent devices with high conformity and excellent light-emitting properties are of particular interest [1,2,3,4]. Such devices would enable a plethora of new applications, such as stretchable displays [5,6,7], electronic textiles [8,9], advanced biomedical imaging and lighting [10], conformable visual readouts in arbitrary shapes [11], and novel health-monitoring devices [12]. For example, Wang and co-workers have developed stretchable electroluminescent devices using ionic conductors [13]. Recently, Larson and co-workers have described a highly-stretchable electroluminescent skin for optical signaling and tactile sensing [14]. Nevertheless, all these pioneering works are geared toward developing stretchable electroluminescent devices based on the structure of their conventional counterparts.

It is highly desirable to develop stretchable and high performance smart devices based on fibers to enable wide usage in smart electronic products. Compared to conventional planar electronic devices, devices based on fibers have been attracting increasing interest due to the beneficial combination of miniaturization, adaptability, and wearability. To date, supercapacitors [15], solar cells [16], triboelectric nanogenerators [17], human health monitors [18], and artificial muscles [19] have been reported as stretchable fiber devices. For example, Yang and co-workers fabricated a highly-stretchable supercapacitor fiber with a tensile strain of 100% [15]. Peng and co-workers developed a stretchable polymer solar cell fiber on an elastic substrate with a spring-like structure [16]. Baughman and co-workers reported highly-stretchable (up to 1320%) sheath-core conducting fibers created by wrapping carbon nanotube sheets oriented in the fiber direction on stretched rubber fiber cores [19]. However, highly-stretchable alternating current electroluminescent (ACEL) fibers, which enable both excellent light-emitting performance and stretchability, are yet to be explored. Challenges in stretchable electroluminescent fiber devices are their requirements in electrode transparency and conductivity and in the stretchability of dielectric materials. Additionally, the electroluminescent phosphors tend to be non-stretchable. Furthermore, difficulties in the complicated fabrication process of devices employing stretchable and fiber structures may be encountered. Moreover, it is important to find an all-encompassing process to fabricate electroluminescent fiber devices that can be woven into various flexible textiles or integrated into soft electronics systems for use in portable light-emitting electronic devices [20].

Here, we present a flexible and stretchable ACEL fiber via an all solution-processed scalable method. The ACEL fiber comprises an elastic core fiber from polydimethyl-siloxane (PDMS), two electrodes (inner and outer) from silver nanowires (AgNWs), and an emissive layer from ZnS:Cu phosphors/PDMS composite. The luminance of the resulted ACEL fiber is largely independent of the stretching strains. Notably, it maintained a stable luminance performance during 6000 stretch-release cycles within a 9% variation. The flexible and stretchable ACEL fiber provides high luminance with low hysteresis and demonstrates excellent mechanical and optical stability during operation.

## 2. Results and Discussion

**Figure 1:**
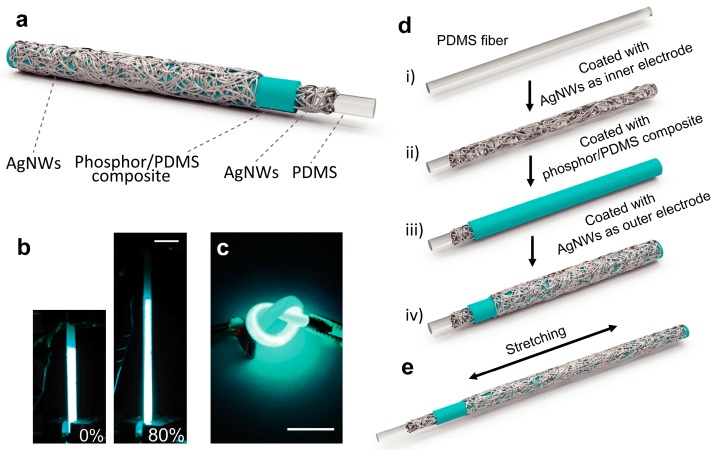
(**a**) Schematic of the structure of the stretchable ACEL fiber. (**b**) Photographs of the stretchable ACEL fiber at original state (left) and at stretched state with 80% strain (right). (**c**) Photograph of the knotted ACEL fiber. All scale bars are 1 cm. (**d**,**e**) Illustration of the fabrication processes of the stretchable ACEL fiber.

Figure 1a demonstrates the configuration of the ACEL fiber device. The detailed fabrication process of our stretchable ACEL fiber is schematically shown in Figure 1d,e. A transparent and elastic PDMS fiber was explored as the stretchable substrate for the ACEL fiber. AgNWs were employed as both the inner and outer electrodes, which can offer a combination of remarkable properties including high optical transparency and electrical conductivity, as well as flexibility and mechanical stability [21,22,23,24]. The AgNWs electrode layers were fabricated by spray-coating, which is easy-to-handle, low-cost, compatible, and scalable [25,26]. In order to obtain a homogeneous and highly-conductive AgNWs film, the PDMS core fiber needed to be heated and rotated along with its longitudinal axis during the spray coating process. An emissive layer consisting a bubble-free and uniform blend of PDMS and ZnS:Cu particles was sandwiched between two AgNWs electrodes. ZnS:Cu is a typical electroluminescent phosphor material that emits light via excitations within intrinsic heterojunctions under an AC electric field. Its emission colors can be easily tuned by doping with different concentrations and types of active elements [14,27]. The emissive layer was dip-coated onto the AgNWs inner electrode. After crosslinking, the electroluminescent layer inherits the excellent stretchability from the PDMS matrix, while sustaining its functionality as emissive material [1]. Additionally, a protecting layer of PDMS was dip-coated onto the whole fiber device to avoid short circuiting and physical damage of the outer AgNWs network during knotting. The resulted electroluminescent fiber was highly flexible and stretchable; it benefited from the elasticity of the PDMS fiber substrate and the ZnS:Cu/PDMS composite layer, as well as from the stable conductivity of the inner and outer AgNW electrodes. As shown in Figure 1b, the ACEL fiber could be stretched up to 80% strain while maintaining its electroluminescent properties. Furthermore, it maintained excellent light-emitting performance during and after being knotted (Figure 1c).

**Figure 2:**
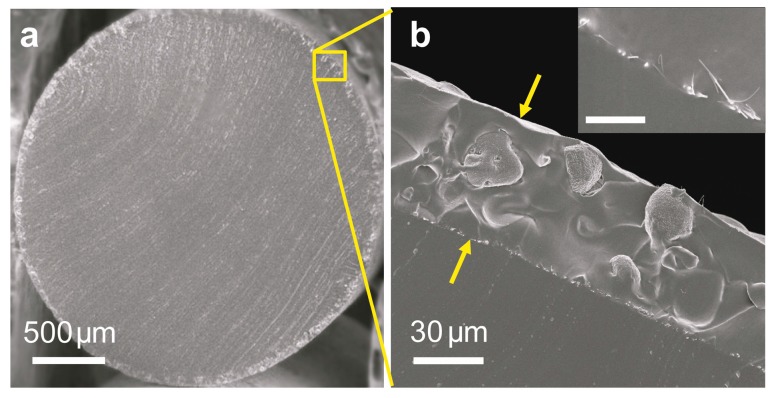

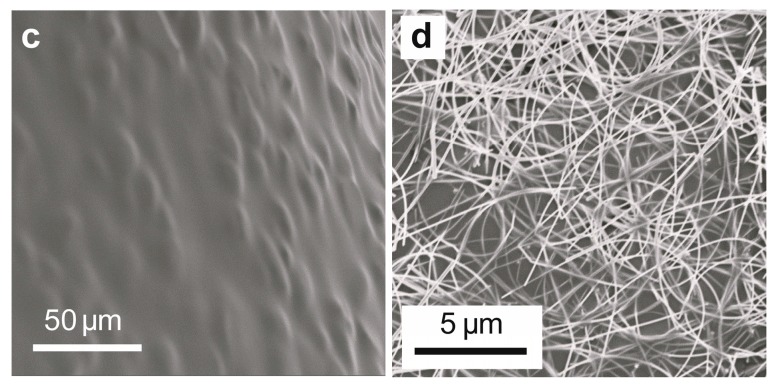
Scanning electron microscope images of the stretchable ACEL fiber. (**a**,**b**) Cross-section images of stretchable ACEL fiber with different magnifications. Inset shows detailed image of AgNWs sandwiched by PDMS and ZnS:Cu/PDMS composite layers. Scale bar is 4 μm. (**c**) Surface morphology of light-emitting layer. (**d**) AgNWs on the ZnS:Cu/PDMS composite surface.

The structures and morphologies of different components of the stretchable ACEL fiber are characterized by scanning electron microscope (SEM). Shown as Figure 2a, the diameter of the core elastic PDMS fiber was ~2.5 mm, which can be controlled by the diameter of the tube template. The total thickness of the active shell of the ACEL fiber, combining both the inner and outer AgNWs electrodes as well as the ZnS:Cu/PDMS composite layer was ~55 μm (Figure 2b). The inset of Figure 2b shows tight contact between AgNW electrode and the core PDMS fiber, as well as between the AgNW electrode and the ZnS:Cu/PDMS composite layer. A rough surface was obtained after coating with the phosphor ZnS:Cu/PDMS composite layer, which could be ascribed to the random stacking of ZnS:Cu particles with diameters in the range of 12–25 μm (Figure 2c). This surface roughness was beneficial for the adhesion of the outer AgNWs electrode layer. Figure 2d shows the homogeneous distribution of AgNWs with an average diameter of ~100 nm, on the ZnS:Cu/PDMS composite surface. The layered device maintained its luminance performance under hundreds of stretching–releasing cycles partially due to the good contact between each layer, as evidenced in Figure 2, without noticeable bubbles or gaps or other areas that failed to bond.

**Figure 3:**
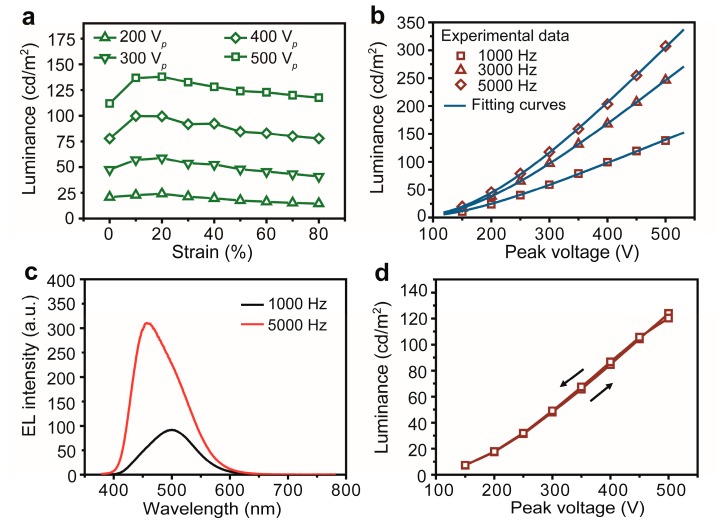
(**a**) Luminance–strain characteristics of the stretchable ACEL fiber under different voltages (1 kHz AC frequency); (**b**) luminance–voltage fitting curves of the stretchable ACEL fiber at 1–5 Hz and 20% strain from experimental data and theoretical calculation; (**c**) electroluminescent spectra of the stretchable ACEL fiber at 1 kHz and 5 kHz AC frequencies; and (**d**) the luminance-voltage hysteresis characteristics of the stretchable ACEL fiber at 50% strain (1 kHz AC frequency).

The dependence of luminance on the stretching strains for the ACEL fiber is shown in Figure 3a. Stable light emission was sustained in the fiber device at up to 80% strain with an applied AC frequency of 1 kHz. At a strain of 20%, a maximum luminance was observed. According to the following equation:

*E* = *U*/*d* (1)

where *E* is the electric field, *U* is the voltage passing through the phosphor layer, and *d* is the thickness of the phosphor layer, the increased luminance could be attributed to the decrease in the thickness of the phosphor ZnS:Cu/PDMS composite layer, resulting in the increase of the electric field (*E*) passing through the phosphor ZnS:Cu/PDMS composite layer. This geometry-induced field gradient led to the acceleration of electrons in phosphor particles and, consequently, increased the luminance of the ACEL fiber. For a further increase of the stretching strain, the luminance dropped gradually, which could due to a further decrease of the density of the phosphor particles. As shown in Figure 3a, under a 500 V bias voltage, the luminance increased to 138.0 cd/m^2^ at a strain of 20%, corresponded with a ~123.3% increase in the luminance value. At the strain of 80%, the luminance dropped to 117.6 cd/m^2^; a value which is still ~5.1% higher than that of the non-stretched fiber. 

Furthermore, we studied the relationship between voltage and luminance at an AC frequency of 1 to 5 kHz and a strain of 20% (Figure 3b). The measured data could be fitted reasonably well with the equation:

*L* = *L*_0_ exp(−β/V^1/2^) (2)

where *L* is the luminance, *V* is the applied voltage, and *L*_0_ and *β* are the constants related to the device. Even under 20% strain and at different frequencies and for various fitting parameters *L*_0_ and *β*, the root-mean-square deviations of the fitting was ~0.999. This observation indicates that, for the stretched ACEL fiber, the emission performances can be well-described with the conventional equation, and the fit still maintains its stability. As shown in Figure 2b, a maximum luminance of 307.3 cd/m^2^ was obtained at 5 kHz and a bias voltage of 500 V. Although the applied voltage of our ACEL fiber device is still high, it can be greatly reduced by fine-tuning the device architecture and material’s properties (such as by reducing thickness of the light-emitting layer, optimizing inorganic light-emitting particle size, and choosing stretchable insulating materials with higher dielectric constant). In Figure 3c, the EL emission spectra is centered at 500 nm with a full-width-at-half-maximum (FWHM) of 97 nm (1 kHz and 500 V*_p_*) and shifted to 457 nm with a FWHM of 97 nm at 5 kHz and a bias voltage of 500 V. At the same time, the Commission Internationale de I’Eclairage (CIE) color coordinate changes from x = 0.18, y = 0.35 to x = 0.16, y = 0.22. This observation indicates a change of the emitting color from green to blue with increasing frequency. This phenomenon is similar to those reported in the literatures [28,29,30]. It is generally accepted that more than one emission band exists in ZnS:Cu phosphors. There is a green emission likely arise from the transition between conduction band and the impurity induced green center of Cu, whereas another blue emission is possibly related to the blue centers. At high excitation frequencies, a blue-colored emission occurs because of the higher excitation energy. While low frequencies are insufficient to activate the blue emission centers, the emission is dominated by lower energy levels of the green centers. Hence, an increase in frequency results in a blue shift in the emission wavelength. 

To evaluate the hysteresis of luminance–voltage behavior and verify the reversible performance of the ACEL fiber after stretch-release cycles, the corresponding luminance for the fiber at 50% strain were recorded with applied bias voltages increasing from 150 to 500 V*_p_*, followed by further stretch the fiber to 80% strain and release it back to 50% strain, and then recording the luminance with applied voltages decreasing from 500 to 150 V*_p_*. We believe that the changes of the thickness of the phosphor layer, the density of the phosphor particles, the conductivity both of the inner and outer AgNWs, and the contact resistance between each layer can all contribute to hysteretic behavior. Notably, hardly any hysteresis was observed in Figure 3d, indicating that the ACEL fibers have excellent stability during the stretch-release cycles.

**Figure 4:**
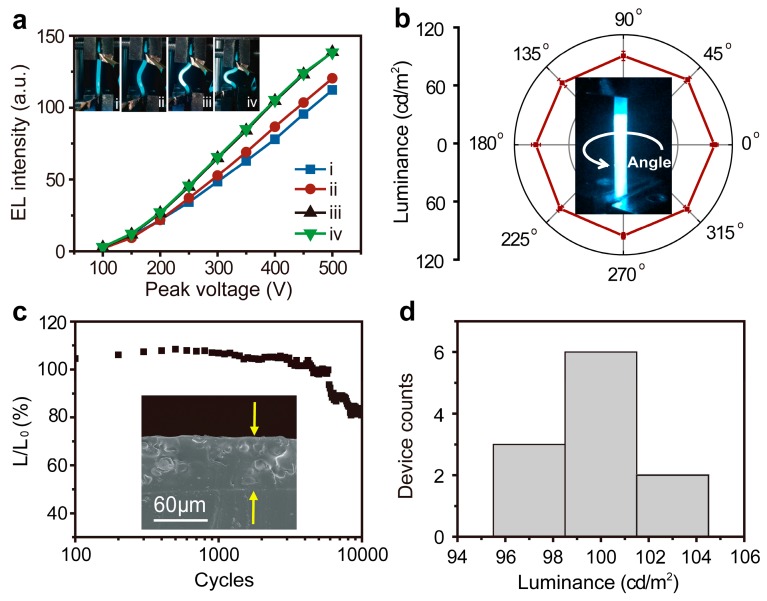
(**a**) Luminance–voltage characteristics of the stretchable ACEL fiber at different bending states (1 kHz AC). The inset photographs are the stretchable ACEL fiber at different bending states. (**b**) Dependence of luminance on viewing angle of a stretchable ACEL fiber at 20% strain (1 kHz AC, 400 V*_p_*). Inset shows the stretchable ACEL fiber at 20% strain. (**c**) Stability test of the stretchable ACEL fiber when mechanically cycled to 50% strain (1 kHz AC, 300 V*_p_*). *L*_0_ and *L* correspond to luminance measured at the first cycle and the current cycle, respectively. The inset SEM image is the cross-section of the stretchable ACEL fiber after 10,000 stretching-releasing cycles. (**d**) The luminance statistics at 20% strain (1 kHz AC, 400 V*_p_*) of eleven as-fabricated ACEL fibers.

The stretchable ACEL fibers also exhibited a good bending stability. A fiber could be bent to different states (Figure 4a) without degrading the luminance. Specifically, the luminance gradually increased as the bending curvature increases and then saturated. In Figure 4a, state iii and state iv show similar light emitting performance. With the advantage of the one-dimensional structure, our fiber device offers light-emission in all directions. As shown in Figure 4b, the luminance is almost independent of the viewing angles, even the luminance between different samples has a slight change. Figure 4c shows the luminance performance of an ACEL fiber which was stretched and released for 10,000 cycles to 50% strain, under a 1 kHz sine-wave 300 V bias voltage. The luminance increases during the 1st to the 300th stretching-releasing cycles is due to the slightly elongation of the elastic PDMS fiber. It did not have enough time to recover back to its 100% original length, resulting in the slightly increased luminance compared to the first cycle of the fiber. The fiber device shows relatively stable performance with a vibration of the luminance less than 9% ((*L* – *L*_0_)/*L*_0_) over the investigated 6000 stretching-releasing cycles. After 6000 cycles, there was an obvious luminance decrease of the fiber device, which may due to the contact resistance increase among the AgNWs of the unencapsulated devices. Additionally, the slight emission fluctuation in the cycles was attributed to the contact problem between the copper foil electrode and AgNWs during the measurement. Even after experiencing 10,000 stretch-release cycles, the cross-section of the ACEL fiber still shows good contact interfaces without any obvious visible cracks and damage (shown as the inset of Figure 4c). The luminance performances of eleven samples were measured, under 1 kHz AC, 400 V*_p_* and 20% strain. Figure 4d shows good reproducibility of the all solution-processed method we used to achieve stretchable ACEL fibers with stable luminance. According to the Shapiro-Wilk test, the *p* value is equal to 0.988 (>0.05), that is, the luminance fitted normal distribution. Over 50% samples exhibits luminance between 98.5 and 101.5 cd/m^2^, and the other data we studied in our work is based on the fiber devices whose luminance performance is around 100 cd/m^2^ under 1 kHz AC, 400 V*_p_*, and 20% strain. Furthermore, the stretchability and light-emitting performance of this ACEL fiber was compared with previously-reported light-emitting fibers (Table 1).

## 3. Materials and Methods 

### 3.1. PDMS Fiber Fabrication

PDMS was prepared from a base polymer and its curing agent (Sylgard 184, Dow Corning, Midland, MI, USA) with the weight ratio of 10:1. The mixture was degassed in vacuum oven for 15 min. The bubbleless mixture was injected into an airtight 2.5-mm-diameter plastic tube template. The mixture was cured at 60 °C for 90 min. Then, the stretchable PDMS fiber was removed from the template. 

### 3.2. Stretchable EL Fiber Fabrication

Starting solution of 10 mg/mL AgNWs ethanol dispersion was obtained from ColdStones Tech. Co. Ltd. (Suzhou, China), wherein the diameters and lengths of the AgNW were 90–110 nm and 30–40 μm, respectively. This solution was diluted to 0.5 mg/mL by adding more ethanol. The AgNWs were spray coated onto PDMS fiber. In order to obtain a homogeneous layer, the PDMS fiber was rotated along with its axis while being heated during spray-coating processes. Electroluminescent phosphor particles (ZnS:Cu) were purchased from Lonco Co. Ltd. (Hong Kong, China) and blended with PDMS at a weight ratio of 2:1. The composite was diluted by adding n-hexane to 25% the weight of the phosphor. The light-emitting layer was obtained by dip coating this diluted composite onto the AgNWs coated fiber. Then the fiber with light-emitting layer was cured at 60 °C for 90 min. Finally, AgNWs were spray-coated as the outer electrode.

### 3.3. Device Characterization

All measurements were performed under ambient conditions. Copper tape was used to electrically connect the AgNW electrodes of the fiber devices to the external power source for further tests. The as-fabricated fiber device was placed in a tensile stage with two clamps which can control the stretch accurately. A function generator (DG4102, Rigol, Beijing, China) connected with a power amplifier (Model 615-10, Trek, Medina, NY, USA) was used to power the as-fabricated fiber device. The electroluminescence emission spectra and luminance were measured by a spectroradiometer (SpectraScan PR-788 with a MS-55 lens, Photo Research, Syracuse, NY, USA). The surface morphology was imaged by SEM measurement (Supra55, Zeiss, Oberkochen, Germany).

## 4. Conclusions

In conclusion, we have reported an alternating current electroluminescent fiber device, which provides unprecedented features, including high stretchability, stability, and excellent light-emitting performance without any noticeable hysteresis, through a simple, scalable, and low-cost fabrication process. In particularly, we found the fabricated fibers provide uniform luminance regardless of viewing angles and maintained a stable electroluminescent performance when subjected to bending, stretching, and knotting. These exceptional features endow our ACEL fiber a luminance of 14.63 cd/m^2^ at 200 V*_p_* and 1 kHz when it was stretched to 80% strain, and a maximum luminance of 307.3 cd/m^2^ at 500 V*_p_* and 5 kHz at 20% strain. Furthermore, the remarkable stability was demonstrated by performing over 6000 stretching-releasing cycles to 50% strain, where the device maintained a stable luminance. In addition, the experimental results fit well with classic theoretical predictions. Due to the easy fabrication process, stable light-emitting performance, and good stretchability of the developed fibers, we believe our stretchable ACEL fiber will conceivably open up new prospective fields and affect various devices encompassing wearable light-emitting electronics, biomedical devices, and other unprecedented applications for the next generation of optoelectronic devices.

## Figures and Tables

**Table 1:** Brief summary of performances reported on light-emitting fibers.

Light-Emitting Layer		Inner/Outer Electrode		Max Luminance (cd/m^2^)	Max Strain (%)	Stretching Cycles	Viewing Angle (°)
Materials	Method	Materials	Method				
PF-B [31]	dip-coating	aligned CNT/aligned CNT	mechanically wrapping/mechanically wrapping	609 (13 V)	0	---	180
Superyellow [32]	dip-coating	PEDOT:PSS/Al/LiF	dip-coating/evaporation	1458.8 (10 V)	0	---	---
α-NPD [33]	evaporation	Al/Al	evaporation/evaporation	---	0	---	---
F8BT [34]	ES ^a)^	liquid metal/ITO	coaxial ES/evaporation/evaporation	2300 (6 V)	0	---	---
[Ru(bpy)_3_]^2+^ (PF_6_^−^)_2_ [35]	ES	ITO/Ca/Al	evaporation/evaporation	---	0	---	---
Graphene QD [36]	drop-casting	---	---	---	0	---	---
ZnS:Cu/PDMS ^b)^	dip-coating	AgNWs/AgNWs	spray-coating/spray-coating	307.3 (500 V*_p_*, 5 kHz)	80	6000	360

^a)^ ES is for electrospinning technique; ^b)^ Our current work.
